# Особенности стероидного профиля при заболеваниях надпочечников у детей

**DOI:** 10.14341/probl13166

**Published:** 2022-12-20

**Authors:** Э. А. Янар, Н. В. Маказан, В. А. Иоутси, М. А. Карева, О. Б. Безлепкина, В. А. Петеркова

**Affiliations:** ФГБУ «Национальный медицинский исследовательский центр эндокринологии» Минздрава России; ФГБУ «Национальный медицинский исследовательский центр эндокринологии» Минздрава России; ФГБУ «Национальный медицинский исследовательский центр эндокринологии» Минздрава России; ФГБУ «Национальный медицинский исследовательский центр эндокринологии» Минздрава России; ФГБУ «Национальный медицинский исследовательский центр эндокринологии» Минздрава России; ФГБУ «Национальный медицинский исследовательский центр эндокринологии» Минздрава России

**Keywords:** мультистероидный профиль, кортикотропинома, болезнь Иценко–Кушинга, кортикостерома, синдром Иценко–Кушинга, инциденталома надпочечника, дети

## Abstract

ВВЕДЕНИЕ. Образования надпочечников часто сопровождаются гиперпродукцией стероидных гормонов, в связи с чем определение их концентрации играет важную роль в дифференциальной диагностике заболеваний надпочечников. Определение уровня стероидных гормонов с помощью тандемной масс-спектрометрии является одним из основных диагностических методов изучения стероидогенеза. На настоящий момент исследование стероидного профиля крови и мочи вызывает особый интерес для дифференциальной диагностики различных видов объемных образований надпочечников.ЦЕЛЬ. Исследование стероидного профиля пациентов детского возраста с патологией надпочечников (инциденталомы, гиперкортицизм центрального и надпочечникового генеза, адренархе).МАТЕРИАЛЫ И МЕТОДЫ. Ретроспективное исследование мультистероидного профиля 41 пациента с патологией надпочечников, наблюдавшихся в период с 2005 г. по 2020 г. в ФГБУ «НМИЦ эндокринологии» Минздрава России.РЕЗУЛЬТАТЫ. Все пациенты были разделены на группы в зависимости от нозологического диагноза: с АКТГ-зависимым гиперкортицизмом (кортикотропиномы) — 7 пациентов, с АКТГ-независимым гиперкортицизмом (кортикостерома) — 4, с инциденталомой надпочечников — 7, с преждевременным адренархе — 23. В группе пациентов с кортикостеромами выявлены статистически значимые более высокие уровни 11-дезоксикортизола (р=0,0035) и более низкие уровни 17-гидроксипрегненолона (р=0,0026) и дегидроэпиандростерона (р=0,0047) по сравнению с другими группами. Статистически значимых различий показателей мультистероидного профиля между другими группами выявлено не было.ЗАКЛЮЧЕНИЕ. По результатам нашей работы исследование мультистероидного профиля может использоваться в качестве дополнительного метода дифференциальной диагностики пациентов с образованиями надпочечников с и без гормональной гиперпродукции (кортикостеромы и инциденталомы надпочечников). Необходим дальнейший поиск стероидных маркеров для дифференциальной диагностики различных заболеваний надпочечников у детей.

## Abstract (en)

BACKGROUND: Adrenocortical adenomas are often followed with steroid hormones hyperproduction, and therefore determination of their concentration plays an important role in the differential diagnosis of adrenal diseases. Steroid profiling by tandem mass spectrometry is one of the main diagnostic methods in steroidogenesis characterization. Currently plasma and urinary steroid profiling is of particular interest in differential diagnosis and subtyping patients with adrenocortical adenomas.AIM: Steroid profiling of pediatric patients with adrenal diseases (incidentalomas, ACTH-secreting pituitary adenoma, ACTH-independent Cushing syndrome, premature adrenarche).MATERIALS AND METHODS: We conducted a retrospective analysis of steroid profile of 41 pediatric patients with adrenal diseases who were observed between 2005 and 2020 at the Endocrinology Research Centre.RESULTS: All patients were divided into groups due to diagnosis: with ACTH-secreting pituitary adenoma [n=7], ACTH-independent Cushing syndrome (autonomous cortisol secretion by an adrenal adenoma) [n=4], with incidentaloma [n=7] and premature adrenarche [n=23]. In group of patients with ACTH-independent Cushing syndrome identified statistically significant higher levels of 11-deoxycortisol (р=0, 0035) and significant lower levels of 17-hydroxypregnenolone (р=0, 0026) and DHEA (р=0, 0047) compared to other groups. Statistically significant differences in steroid profiles between other groups were not identified.CONCLUSION: Results of our study steroid profiling can be used as additional differential diagnosis method in patients with adrenocortical adenomas with or without hormonal hyperproduction (ACTH-independent Cushing syndrome and incidentaloma). Further studies are needed to identify steroid markers for subtyping pediatric adrenal diseases.

## ВВЕДЕНИЕ

Продукция стероидных гормонов корой надпочечников представляет собой сложный каскад реакций, конечными продуктами которых являются альдостерон, кортизол и андрогены [1] (рис. 1). Опухоли надпочечников часто сопровождаются автономной гиперсекрецией различных стероидных гормонов и/или их предшественников [2], в связи с чем определение их концентрации играет важную роль в дифференциальной диагностике заболеваний надпочечников.

**Figure:**
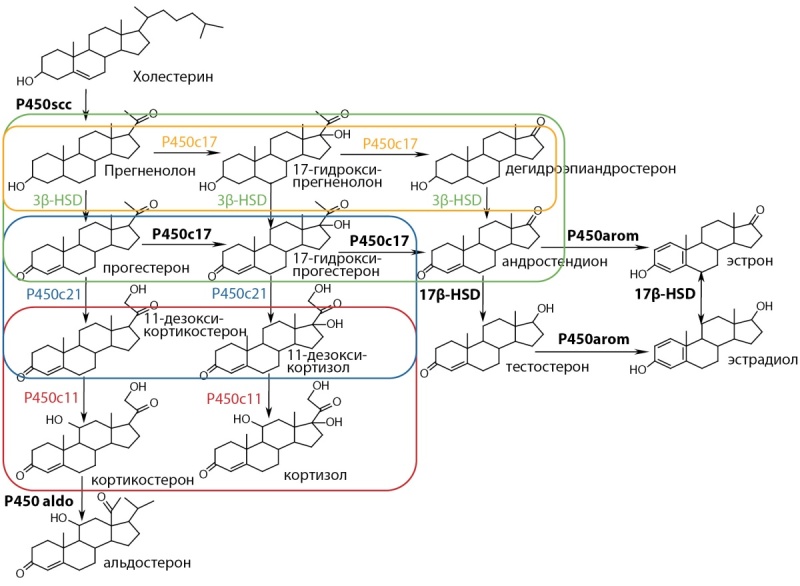
Рисунок 1. Схема стероидогенеза.Figure 1. Scheme of steroidogenesis Примечания: Р450scc — 20,22-десмолаза; Р450с17 — 17α-гидроксилаза; 3β-HSD — 3β-гидроксистероиддегидрогеназа; P450c21 — 21-гидроксилаза; P450c11 — 11β-гидроксилаза; P450aldo — альдостеронсинтаза; 17β- HSD — 17β-гидроксистероиддегидрогеназа; P450arom — ароматаза. Желтым цветом выделена область активности фермента 17α-гидроксилазы/17, 20-лиазы; зеленым цветом — 3β-гидроксистероиддегидрогеназы; синим цветом — 21-гидроксилазы, красным цветом — 11β-гидроксилазы.

В настоящее время одними из ведущих методов диагностики являются иммунологические методы, основанные на взаимодействии антигенов и антител, которые используются для определения уровня гормонов и множества других показателей [3]. Данный метод исследования обладает высокой чувствительностью и специфичностью, однако имеет несколько существенных недостатков при патологии надпочечников: определение неправильной концентрации определяемого стероида из-за наличия перекрестных реакций меченых антител с соединениями, близкими к нему по строению; невозможность определить одновременно несколько стероидов; отсутствие антисывороточных антигенов ко всем метаболитам стероидов [4].

Масс-спектрометрия (МС) представляет собой физико-химический метод анализа, в основе которого лежат генерирование ионов из молекул или атомов исследуемых веществ и разделение их по величинам отношения массы иона к его заряду в электрических или электрических и магнитных полях. Этот метод позволяет сделать вывод о молекулярной массе исследуемого вещества, его составе и структурных особенностях [5]. В сочетании с газовой или высокоэффективной жидкостной хроматографией (ГХ-МС и ВЭЖХ-МС) МС становится методом количественного анализа сложных смесей. Так, метод ГХ-МС получил широкое распространение, в том числе для определения стероидного профиля мочи [6–8]. Данный диагностический метод устраняет недостатки иммунологических методов исследования, однако требует существенных временных затрат, связанных со сложным и трудоемким процессом пробоподготовки, длительным аналитическим циклом для каждого образца и весьма трудоемким процессом обработки полученных данных [7]. Метод определения стероидных гормонов с помощью ВЭЖХ с тандемным МС-детектированием (ВЭЖХ-МС/МС) в настоящее время является одним из основных диагностических методов изучения стероидогенеза [9][10] и играет важную роль в диагностике различных заболеваний надпочечников, таких как врожденная дисфункция коры надпочечников, первичная надпочечниковая недостаточность и первичный гиперальдостеронизм. Этот метод одинаково пригоден для анализа как мочи, так и сыворотки или плазмы крови [10][11]. На настоящий момент исследование стероидного профиля крови и мочи вызывает особый интерес для дифференциальной диагностики различных видов объемных образований надпочечников, исключения и уточнения генеза эндогенного гиперкортицизма.

Цель — исследование стероидного профиля пациентов детского возраста с патологией надпочечников (инциденталомы, гиперкортицизм центрального и надпочечникового генеза, адренархе).

## МАТЕРИАЛЫ И МЕТОДЫ

## Место и время проведения исследования

Место проведения. ФГБУ «Национальный медицинский исследовательский центр эндокринологии» Минздрава России, Москва, Россия.

Время исследования. Период с 2005 по 2020 г.

## Изучаемые популяции (одна или несколько)

Популяция: одна.

Критерии включения: возраст на момент установления диагноза менее 18 лет; подтвержденный диагноз адренархе, инциденталомы надпочечника, эндогенного гиперкортицизма центрального и надпочечникового генеза.

Критерии исключения: другая гиперсекреция надпочечников (альдостеромы, феохромоцитомы), отсутствие данных мультистероидного профиля.

## Способ формирования выборки из изучаемой популяции (или нескольких выборок из нескольких изучаемых популяций)

Сплошной.

## Дизайн исследования

Одноцентровое одномоментное ретроспективное исследование.

## Описание медицинского вмешательства (для интервенционных исследований)

Для анализа использованы данные историй болезни пациентов. Все медицинские вмешательства проводились вне исследования в рамках рутинной клинической практики по актуальным на соответствующий момент времени международным стандартам и строго при наличии показаний у каждого конкретного пациента.

## Методы

Диагноз эндогенного гиперкортицизма подтверждался на основании 2 положительных лабораторных тестов из 3: высокий уровень кортизола вечером (>300 нмоль/л), подтверждающий нарушение ритма секреции кортизола, повышенный уровень кортизола в суточной моче (>400 нмоль/сут), отсутствие подавления кортизола после ночного дексаметазонового теста или малой дексаметазоновой пробы (ночной тест — 1 мг на ночь, малый тест с дексаметазоном — 30 мкг/кг (максимально 0,5 мг) через каждые 6 ч в течение 48 ч).

Для дифференциальной диагностики между АКТГ-зависимым и АКТГ-независимым гиперкортицизмом проводился контроль уровня АКТГ сыворотки крови. При выявлении уровня АКТГ менее 5 пг/мл устанавливался диагноз АКТГ-независимого гиперкортицизма надпочечникового генеза. При выявлении нормального или высокого уровня АКТГ утром (≥60 пг/мл) проводилась большая дексаметазоновая проба (120 мкг/кг массы тела (максимально 2 мг) каждые 6 ч в течение 48 ч) для дифференциальной диагностики между кортикотропиномой и АКТГ-эктопированным синдромом. Подавление уровня кортизола более чем на 50% исходного рассматривалось как подтверждение центрального генеза АКТГ-зависимого гиперкортицизма.

Для топической диагностики проводилась МРТ головного мозга с контрастным усилением при подтвержденном центральном генезе АКТГ-зависимого гиперкортицизма, КТ или МРТ надпочечников с контрастным усилением при АКТГ-независимом генезе гиперкортицизма.

Диагноз инциденталомы надпочечника устанавливался при случайно выявленном образовании надпочечника на основании исключения гормональной гиперпродукции и определения злокачественного потенциала опухоли. Эндогенный гиперкортицизм был исключен на основании тестов с дексаметазоном (см. выше). Для исключения гиперсекреции катехоламинов производился сбор суточного анализа на уровни метанефринов и норметанефринов, диагноз исключался при уровнях метанефринов и норметанефринов, не превышающих верхних пределов нормы (312 мкг/сут и 445 мкг/сут соответственно). Первичный альдостеронизм исключался на основании отсутствия артериальной гипертензии и нормального уровня альдостерон-ренинового соотношения. Гиперандрогения исключалась при отсутствии клинических проявлений и нормальных уровней андрогенов сыворотки для пола и возраста пациента.

Для определения злокачественного потенциала опухоли пациентам проводилась КТ надпочечников с оценкой нативной плотности образования и плотности в артериальную, венозную и отсроченную фазы контрастирования. Злокачественный потенциал опухоли оценивался как высокий при выявлении высокой нативной плотности образования (>10–15 ед.Н.) и задержки вымывания контраста в отсроченную фазу (<50% через 10 мин). Пациенты с высоким злокачественным потенциалом были исключены по причине малочисленности группы — 2 человека.

Группа пациентов с преждевременным адренархе была выбрана как группа сравнения, учитывая тот факт, что клинические проявления преждевременного пубархе входят в дифференциально-диагностический ряд как объемных образований надпочечников, так и гиперкортицизма.

В мультистероидный профиль входили следующие показатели: прогестерон (n=40), 17-гидроксипрегненолон (n=37), дегидроэпиандростерон (n=41), 17-гидроксипрогестерон (n=41), альдостерон (n=37), 11-дезоксикортизол (n=41), 21-дезоксикортизол (n=41), дезоксикортикостерон (n=34), кортизон (n=24), прегненолон (n=24), андростендион (n=41), кортикостерон (n=40), тестостерон (n=41), кортизол (n=41). Показатели андростендиона и тестостерона были исключены из исследования в связи с невозможностью разделения групп и выраженным различием показателей в зависимости от возраста и пола пациентов.

## Статистический анализ

Статистическая обработка полученных данных проводилась с использованием пакета статистических программ Statistica 13. Распределения количественных признаков представлены медианами (Me) и интерквартильными интервалами [ Q1; Q3]. Для сравнения групп использовался тест Краскела–Уоллиса. Пороговым уровнем статистической значимости Р считали 0,05. Для нивелирования проблемы множественных сравнений применяли поправку Бонферрони.

## Этическая экспертиза

Протокол исследования был одобрен на заседании локального этического комитета ФГБУ «НМИЦ эндокринологии» Минздрава России (протокол №18 от 24.10.2018 г.).

## РЕЗУЛЬТАТЫ

В исследование был включен 41 пациент с патологией надпочечников, наблюдавшийся в период с 2005 по 2020 г. в ФГБУ «НМИЦ эндокринологии» Минздрава России. Все пациенты были разделены на 4 группы в зависимости от нозологического диагноза: 1-я группа — пациенты с АКТГ-зависимым гиперкортицизмом (кортикотропиномы; n=7), 2-я группа — пациенты с АКТГ-независимым гиперкортицизмом (кортикостерома; n=4), 3-я группа — пациенты с инциденталомой надпочечников (n=7), 4-ягруппа — пациенты с преждевременным адренархе (n=23).

В 1-й и 2-й группах всем пациентам проведено хирургическое лечение с последующим патоморфологическим исследованием удаленных образований. В 1-й группе пациентов патоморфологически был подтвержден диагноз кортикотропиномы, во 2-й группе у одного пациента выявлена микронодулярная гиперплазия коры надпочечника, у оставшихся пациентов — кортикостерома надпочечника.

Хирургического лечения и патоморфологического исследования в группе пациентов с инциденталомами надпочечников не проводилось.

Клинико-лабораторная характеристика пациентов представлена в таблице 1.

**Table:** Таблица 1. Характеристика групп пациентовTable 1. Characteristics of patient groups Примечание. Описательная статистика количественных признаков представлена медианами, интерквартильными интервалами — Me [ Q1; Q3]. Здесь и в таблицах 2 и 3: БИК — болезнь Иценко–Кушинга; СИК — синдром Иценко–Кушинга.

	Группа 1(пациенты с БИК) N=7	Группа 2(пациенты с СИК) N=4	Группа 3 (пациенты с инциденталомой) N=7	Группа 4(пациенты с адренархе) N=23
Возраст на момент обследования, лет	10,3 [ 7,15; 17]	15,5 [ 8,375; 16,55]	15,67 [ 10,61; 16,7]	7,25 [ 6,6; 8,42]
SDS роста	-0,06 [ -0,88; 0,59]	-1,53[ -2,49; -0,15]	-0,22[ -0,31; 1,11]	1,25[ 0,82; 1,77]
SDS ИМТ	2,46 [ 1,17; 3,52]	0,045 [ -0,55; 1,88]	0,27 [ -0,94; 0,45]	0,7 [ -0,24; 1,74]
АКТГ (утро), пг/мл	55,3 [ 45,38; 73,04]	1,3 [ 1,215; 1,425]	40,56 [ 21,9; 59,2]	22,91 [ 18,18; 24,98] N=21
Кортизол (утро), нмоль/л	816 [ 673,4; 970]	915,15 [ 609,85; 1099,5]	545,5 [ 356,8; 908,1]	511,5 [ 376,45; 547,85] N=12
Кортизол после малой пробы с дексаметазоном, нмоль/л	99,84 [ 84,4; 257,2]	520,2 [ 73; 1062] N=3	23,85 [ 14,67; 42,12]	
ДГЭА-С, мкмоль/л	5,36 [ 3,87; 7,26] N=4	0,945 [ 0,818; 1,49]	6,75 [ 2,27; 9,31]	3,395 [ 2,33; 5,05] N=22

При анализе мультистероидного профиля были выявлены статистически значимые более высокие уровни 11-дезоксикортизола в группе пациентов с кортикостеромами (р=0,0035). Уровни 11-дезоксикортизола в группе пациентов скортикотропиномами статистически значимо не отличались от групп с инциденталомами надпочечников и адренархе (рис. 2).

**Figure:**
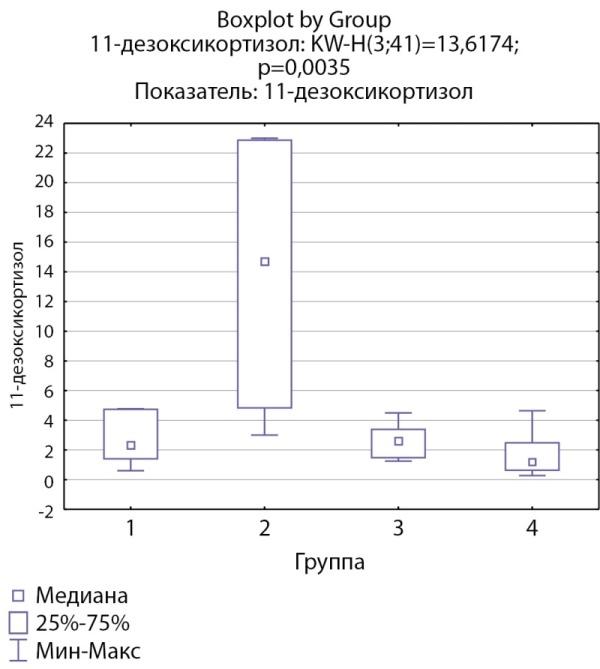
Рисунок 2. Распределение уровней 11-дезоксикортизола по группам: 1-я группа — пациенты с АКТГ-зависимым гиперкортицизмом (кортикотропиномы; n=7), 2-я группа — пациенты с АКТГ-независимым гиперкортицизмом (кортикостерома; n=4), 3-я группа — пациенты с инциденталомой надпочечников (n=7), 4-я группа — пациенты с преждевременным адренархе (n=23).Figure 2. Distribution of 11-deoxycortisol levels by group: group 1 — patients with ACTH-dependent hypercorticism (corticotropinomas; n=7), group 2 — patients with ACTH-independent hypercorticism (corticosteroma; n=4), group 3 — patients with adrenal incidentaloma (n= 7), group 4 — patients with premature adrenarche (n=23)

По показателям 17-гидроксипрегненолона при сравнении между группами отмечаются статистически значимые более низкие уровни данного показателя в группе кортикостером при сравнении с группой инциденталом надпочечника (р=0,0026). Уровни 17-гидроксипрегненолона в группе пациентов с кортикотропиномами статистически значимо не отличались от группы пациентов с адренархе, самые высокие уровни данного показателя отмечены в группе пациентов с инциденталомами надпочечников (рис. 3).

**Figure:**
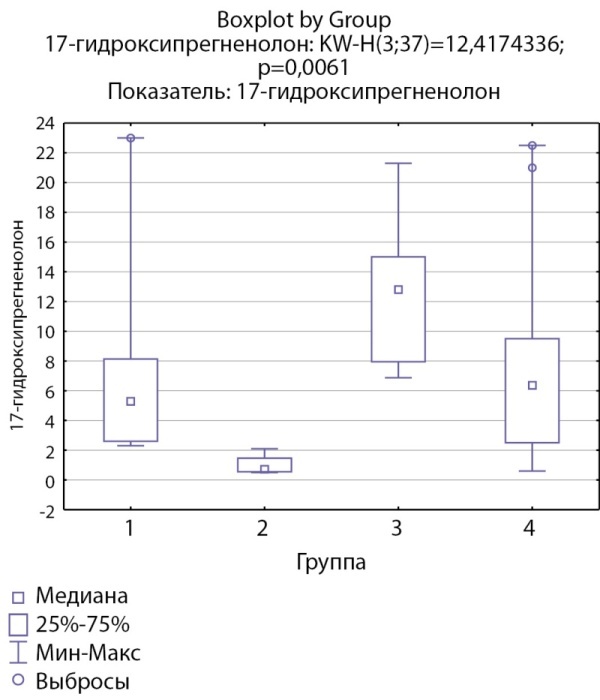
Рисунок 3. Распределение уровней 17-гидроксипрегненолона по группам: 1-я группа — пациенты с АКТГ-зависимым гиперкортицизмом (кортикотропиномы; n=7), 2-я группа — пациенты с АКТГ-независимым гиперкортицизмом (кортикостерома; n=4), 3-я группа — пациенты с инциденталомой надпочечников (n=7), 4-я группа — пациенты с преждевременным адренархе (n=19).Figure 3. Distribution of 17-hydroxypregnenolone levels by groups: group 1 — patients with ACTH-dependent hypercorticism (corticotropinomas; n=7), group 2 — patients with ACTH-independent hypercorticism (corticosteroma; n=4), 3 group — patients with adrenal incidentaloma (n=7), group 4 — patients with premature adrenarche (n=19).

Аналогичный результат получен при оценке уровня дегидроэпиандростерона (ДГЭА) в группах. В группе пациентов с кортикостеромами отмечаются статистически значимо более низкие уровни ДГЭА по сравнению с группой инциденталом надпочечника (р=0,0047) (рис. 4).

**Figure:**
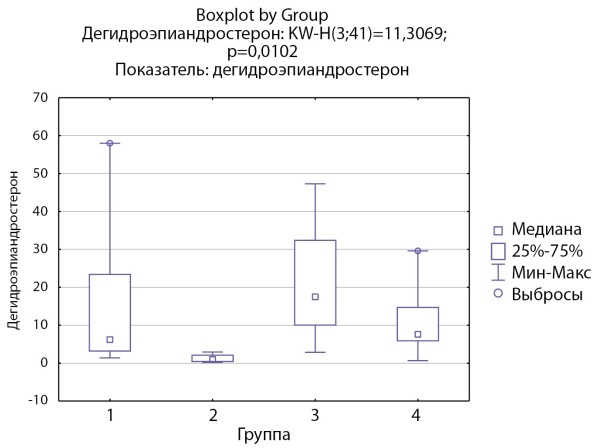
Рисунок 4. Распределение уровней дегидроэпиандростерона по группам: 1-я группа — пациенты с АКТГ-зависимым гиперкортицизмом (кортикотропиномы; n=7), 2-я группа — пациенты с АКТГ-независимым гиперкортицизмом (кортикостерома; n=4), 3-я группа — пациенты с инциденталомой надпочечников (n=7), 4-я группа — пациенты с преждевременным адренархе (n=23).Figure 4. Distribution of dehydroepiandrosterone levels by groups: group 1 — patients with ACTH-dependent hypercorticism (corticotropinomas; n=7), group 2 — patients with ACTH-independent hypercorticism (corticosteroma; n=4), group 3 group — patients with adrenal incidentaloma (n=7), group 4 — patients with premature adrenarche (n=23).

Все показатели мультистероидного профиля приведены в таблице 2.

**Table:** Таблица 2. Стероидный профиль групп пациентовTable 2. Steroid profile of patient groups Примечание. Описательная статистика количественных признаков представлена медианами, интерквартильными интервалами — Me [ Q1; Q3].Для сравнения групп использовался тест Краскела–Уоллиса. Пороговый Р=0,007 (после применения поправки Бонферрони).

Показатель	Группа 1(пациенты с БИК) N=7 (6,5–17,6 года)	Группа 2(пациенты с СИК) N= 4 (1,95–16,9 года)	Группа 3(­пациенты с инциденталомой) N=7 (9,5–17 лет)	Группа 4(пациенты с адренархе) N=23 (3,7–9,0 года)	Р
Прогестерон, нмоль/л	0,14 [ 0,1; 0,3]	0,45 [ 0,055; 13,95]	0,4 [ 0,186; 0,64]	0,275 [ 0,2; 0,47] N=22	0,31
17-гидроксипрегненолон, нмоль/л	5,29 [ 2,6; 8,14]	0,71 [ 0,55; 1,46]	12,8 [ 7,95; 15]	6,36 [ 2,5; 9,5] N=19	0,006 Р1–2=0,21 Р1–3=0,57 Р2–3=0,0026 Р1–4=1,00 Р2–4=0,057 Р3–4=0,47
Дегидроэпиандростерон, нмоль/л	6,2 [ 3,2; 23,4]	1 [ 0,45; 2,13]	17,5 [ 10,06; 32,4]	7,62 [ 5,9; 14,7]	0,01 Р1–2=0,14 Р1–3=1,0 Р2–3=0,0047 Р1–4=1,00 Р2–4=0,052 Р3–4=0,69
17-OH прогестерон, нмоль/л	1,2 [ 0,546; 1,8]	2,2 [ 1; 10,3]	2,7 [ 1,8; 4,05]	0,79 [ 0,6; 1,79]	0,02
Альдостерон, пмоль/л	104 [ 76,7; 150]	70 [ 40; 474]	275 [ 110; 410]	220 [ 100; 420] N=19	0,079
11-дезоксикортизол, нмоль/л	2,3 [ 1,42; 4,75]	14,69 [ 4,84; 22,85]	2,6 [ 1,5; 3,4]	1,2 [ 0,65; 2,49]	0,0035 Р1–2=0,51 Р1–3=1,0 Р2–3=0,77 Р1–4=0,55 Р2–4=0,0053 Р3–4=0,29
21-дезоксикортизол, нмоль/л	0,118 [ 0,01; 0,3]	0,085 [ 0,04; 0,395]	0,02 [ 0,01; 0,43]	0,01 [ 0,01; 0,1]	0,3
Дезоксикортикостерон, нмоль/л	0,2 [ 0,1; 0,3]	0,505 [ 0,3; 1,105]	0,185 [ 0,1; 0,46] N=6	0,1 [ 0,01; 0,34] N=17	0,24
Кортизон, нмоль/л	86,55 [ 80; 89,1] N=6	75 [ 72,8; 120] N=3	73 [ 72,2; 76] N=5	69,7 [ 68; 79] N=10	0,32
Прегненолон, нмоль/л	1,35 [ 0,573; 5] N=6	0,625 [ 0,6; 2,1] N=6	2,44 [ 2,05; 2,63] N=5	3,4 [ 1,4; 4,6] N=10	0,45
Кортикостерон, нмоль/л	9,65 [ 3,5; 11]	4,3 [ 2,69; 12,4]	11,3 [ 8,1; 73,1]	5,735 [ 3,1; 15] N=22	0,09
Кортизол, нмоль/л	486 [ 405; 610]	695 [ 470,5; 780]	403 [ 320; 670]	250 [ 191; 400]	0,0022

Для оценки степени активности ферментов стероидогенеза проводилось сравнение соотношений стероидов к их предшественнику (рис. 1).

Соотношение кортизол/11-дезоксикортизол и кортикостерон/дезоксикортикостерон ниже в группе пациентов с кортикостеромами, что может свидетельствовать о снижении активности 11-бета-гидроксилазы. Статистически значимых различий соотношений кортизол/11-дезоксикортизол и кортикостерон/дезоксикортикостерон в других группах не выявлено (рис. 5 и 6).

**Figure:**
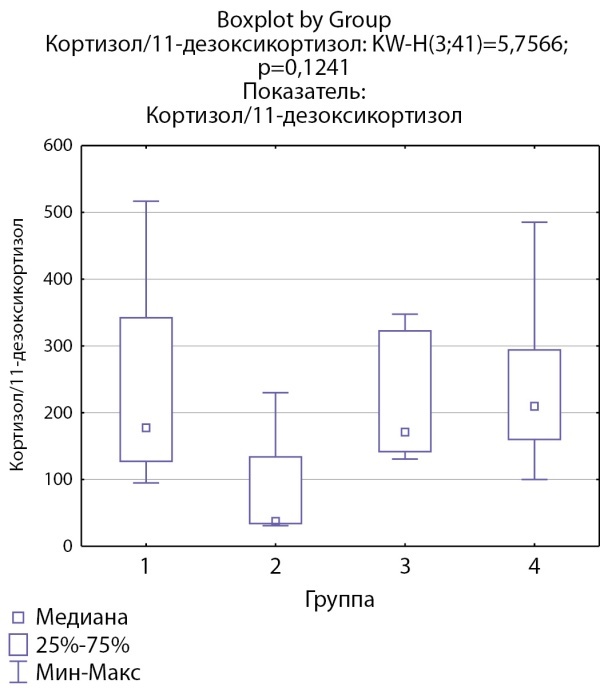
Рисунок 5. Соотношение кортизол/11-дезоксикортизол по группам: 1-я группа — пациенты с АКТГ-зависимым гиперкортицизмом (кортикотропиномы; n=7), 2-я группа — пациенты с АКТГ-независимым гиперкортицизмом (кортикостерома; n=4), 3-я группа — пациенты с инциденталомой надпочечников (n=7), 4-я группа — пациенты с преждевременным адренархе (n=23).Figure 5. Cortisol/11-deoxycortisol ratio by groups: group 1 — patients with ACTH-dependent hypercorticism (corticotropinomas; n=7), group 2 — patients with ACTH-independent hypercorticism (corticosteroma; n=4), group 3 — patients with adrenal incidentaloma (n=7), group 4 — patients with premature adrenarche (n=23).

**Figure:**
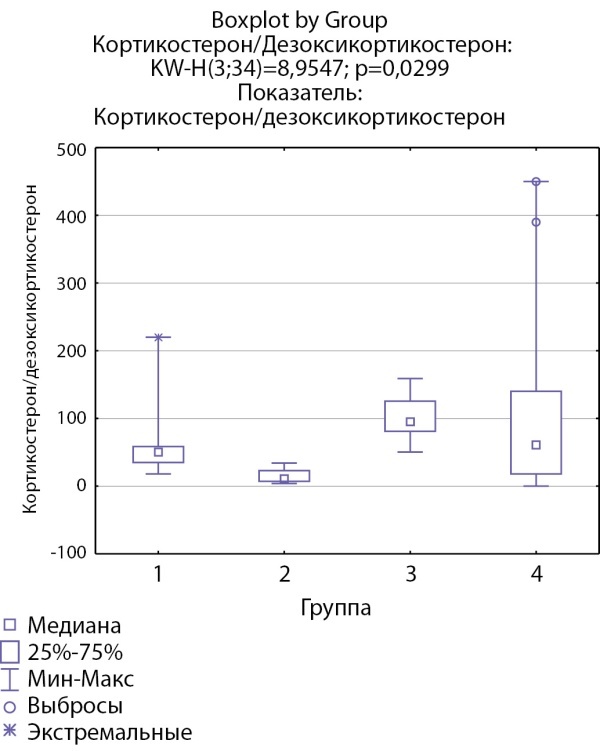
Рисунок 6. Соотношение кортикостерон/дезоксикортикостерон по группам: 1-я группа — пациенты с АКТГ-зависимым гиперкортицизмом (кортикотропиномы; n=7), 2-я группа — пациенты с АКТГ-независимым гиперкортицизмом (кортикостерома; n=4), 3-я группа — пациенты с инциденталомой надпочечников (n=6), 4-я группа — пациенты с преждевременным адренархе (n=17).Figure 6. Corticosterone / deoxycorticosterone ratio by groups: group 1 — patients with ACTH-dependent hypercorticism (corticotropinomas; n=7), group 2 — patients with ACTH-independent hypercorticism (corticosteroma; n=4), 3- group 1 — patients with adrenal incidentaloma (n=6), group 4 — patients with premature adrenarche (n=17).

Соотношения 11-дезоксикортизол/17-гидроксипрогестерон и дезоксикортикостерон/прогестерон, свидетельствующие об активности фермента 21-гидроксилазы, ниже в группах пациентов с инциденталомами надпочечников и адренархе посравнению с группами с гиперкортицизмом различного генеза (рис. 7, 8).

**Figure:**
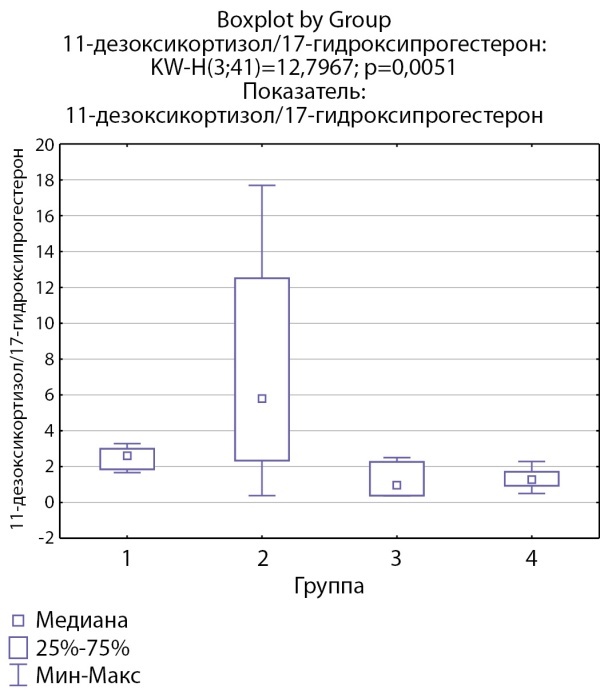
Рисунок 7. Соотношение 11-дезоксикортизол/17-гидроксипрогестерон по группам: 1-я группа — пациенты с АКТГ-зависимым гиперкортицизмом (кортикотропиномы; n=7), 2-я группа — пациенты с АКТГ-независимым гиперкортицизмом (кортикостерома; n=4), 3-я группа — пациенты с инциденталомой надпочечников (n=7), 4-я группа — пациенты с преждевременным адренархе (n=23).Figure 7. 11-deoxycortisol/17-hydroxyprogesterone ratio by groups: group 1 — patients with ACTH-dependent hypercorticism (corticotropinomas; n=7), group 2 — patients with ACTH-independent hypercorticism (corticosteroma; n=4 ), group 3 — patients with adrenal incidentaloma (n=7), group 4 — patients with premature adrenarche (n=23).

**Figure:**
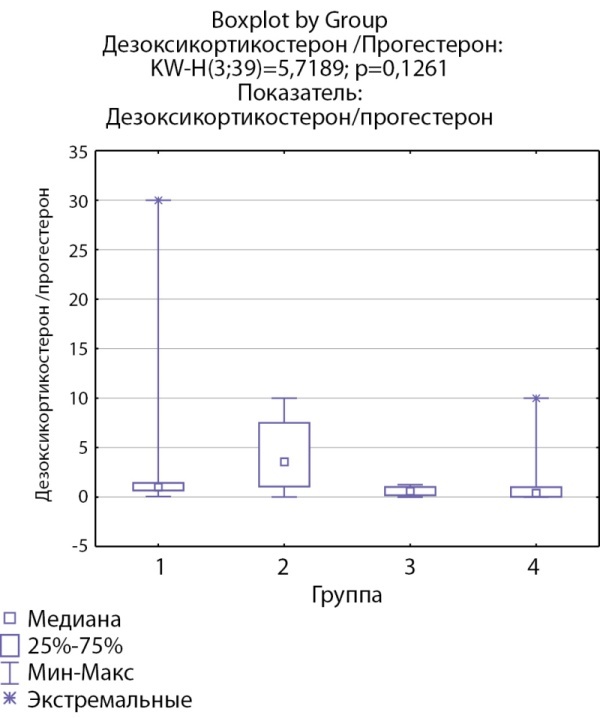
Рисунок 8. Соотношение дезоксикортикостерон/прогестерон по группам: 1-я группа — пациенты с АКТГ-зависимым гиперкортицизмом (кортикотропиномы; n=7), 2-я группа — пациенты с АКТГ-независимым гиперкортицизмом (кортикостерома; n=4), 3-я группа — пациенты с инциденталомой надпочечников (n=7), 4-я группа — пациенты с преждевременным адренархе (n=21).Figure 8. The ratio of deoxycorticosterone/progesterone by groups: group 1 — patients with ACTH-dependent hypercorticism (corticotropinomas; n=7), group 2 — patients with ACTH-independent hypercorticism (corticosteroma; n=4), 3- group 1 — patients with adrenal incidentaloma (n=7), group 4 — patients with premature adrenarche (n=21).

При оценке ферментативной активности 17α-гидроксилазы выявлено, что соотношение 17-гидроксипрегненолон/прегненолон статистически значимо ниже в группе пациентов с кортикостеромами по сравнению с другими группами (рис. 9), статистически значимых различий соотношения ДГЭА/17-гидроксипрегненолон между группами не выявлено. Также выявлено статистически значимое повышение соотношения 17-гидроксипрогестерон/17-гидроксипрегненолон в группе пациентов скортикостеромами, что, возможно, косвенно связано со сниженной секрецией 17-гидроксипрегненолона вследствие низкой 17α-гидроксилазной активности (рис. 10).

**Figure:**
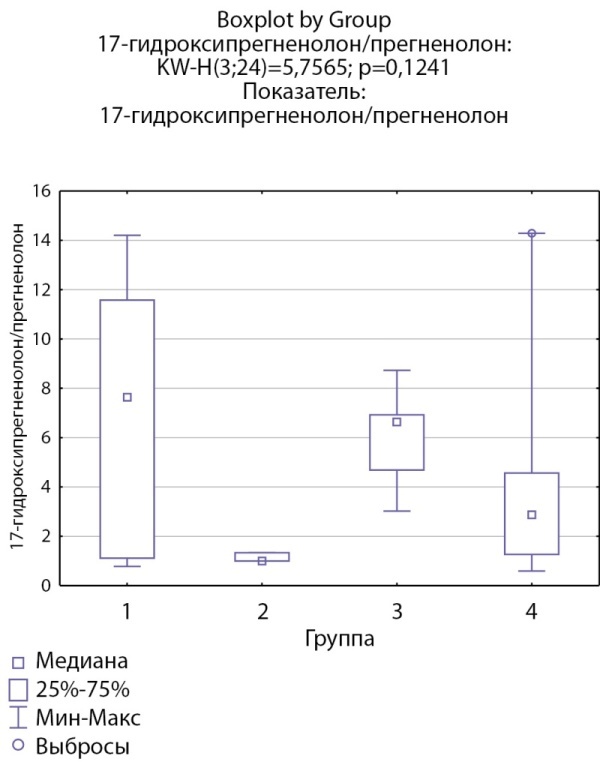
Рисунок 9. Соотношение 17-гидроксипрегненолон/прегненолон по группам: 1-я группа — пациенты с АКТГ-зависимым гиперкортицизмом (кортикотропиномы; n=6), 2-я группа — пациенты с АКТГ-независимым гиперкортицизмом (кортикостерома; n=3), 3-я группа — пациенты с инциденталомой надпочечников (n=5), 4-я группа — пациенты с преждевременным адренархе (n=10).Figure 9. The ratio of 17-hydroxypregnenolone/pregnenolone by groups: group 1 — patients with ACTH-dependent hypercorticism (corticotropinomas; n=6), group 2 — patients with ACTH-independent hypercorticism (corticosteroma; n=3), group 3 — patients with adrenal incidentaloma (n=5), group 4 — patients with premature adrenarche (n=10).

**Figure:**
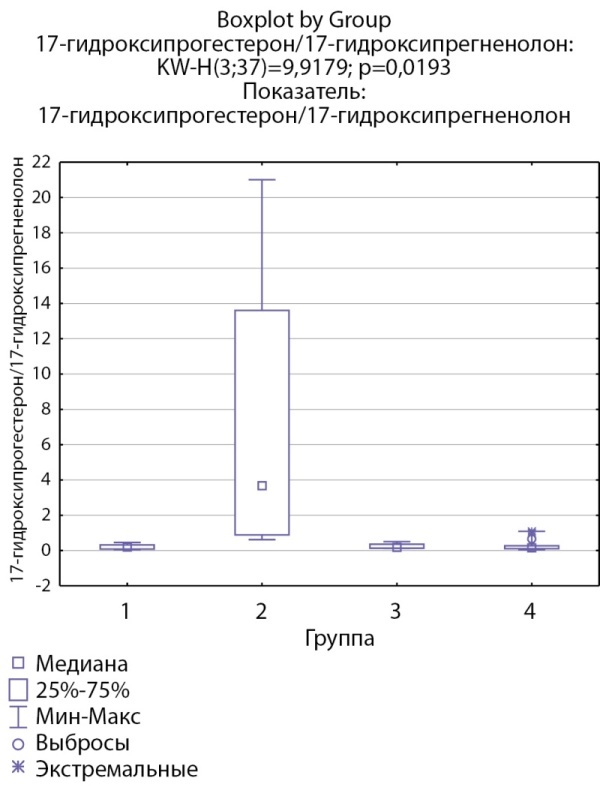
Рисунок 10. Соотношение 17-гидроксикопрогестерон/17-гидроксипрегненолон по группам: 1-я группа — пациенты с АКТГ-зависимым гиперкортицизмом (кортикотропиномы; n=7), 2-я группа — пациенты с АКТГ-независимым гиперкортицизмом (кортикостерома; n=4), 3-я группа — пациенты с инциденталомой надпочечников (n=7), 4-я группа — пациенты с преждевременным адренархе (n=19).Figure 10. 17-hydroxyprogesterone/17-hydroxypregnenolone ratio by groups: group 1 — patients with ACTH-dependent hypercorticism (corticotropinomas; n=7), group 2 — patients with ACTH-independent hypercorticism (corticosteroma; n=4 ), group 3 — patients with adrenal incidentaloma (n=7), group 4 — patients with premature adrenarche (n=19).

Статистически значимые различия в некоторых группах не вычислены в связи с малым количеством пациентов в группах. Все показатели представлены в таблице 3.

**Table:** Таблица 3. Сравнительная оценка соотношений стероидов к их предшественникуTable 3. Comparative evaluation of the ratios of steroids to their predecessor Примечание. Описательная статистика количественных признаков представлена медианами, интерквартильными интервалами — Me [ Q1; Q3].Для сравнения групп использовался тест Краскела–Уоллиса. Пороговый Р=0,007 (после применения поправки Бонферрони).

Показатель	Группа 1(пациенты с БИК) N=7 (6,5–17,6 года)	Группа 2(пациенты с СИК) N= 4 (1,95–16,9 года)	Группа 3(пациенты с инциденталомой) N=7 (9,50–17 лет)	Группа 4(пациенты с адренархе) N=23 (3,7–9,0 года)	Р
Кортизол/11-дезоксикортизол	177,42 [ 127,083; 342,25]	37,48 [ 34,114; 133,78]	170,88 [ 141,83; 322,4]	209,89 [ 160; 294,12]	p=0,12
Кортикостерон/дезоксикортикостерон	50,3 [ 35; 58,485]	10,96 [ 7,14; 22,76]	95,125 [ 80,873; 125,5] N=6	61 [ 18; 140] N=17	p=0,029 Р1–2=0,3 Р1–3=1,0 Р1–4=1,0 Р2–3=0,02 Р2–4=0,08 Р3–4=1,0
Прогестерон/прегненолон	0,155 [ 0,059; 0,24] N=6	21,6 [ 0,0167; 43,36] N=2	0,096 [ 0,09; 0,23] N=5	0,088 [ 0,0378; 0,18] N=10	p=0,97
17-гидроксипрогестерон/17-гидроксипрегненолон	0,21 [ 0,087; 0,316]	3,683 [ 0,893; 13,6]	0,18 [ 0,14; 0,36]	0,16 [ 0,1097; 0,27] N=19	p=0,019 Р1–2=0,036 Р1–3=1,0 Р1–4=1,0 Р2–3=0,14 Р2–4=0,013 Р3–4=1,0
11–дезоксикортизол/17-гидроксипрогестерон	2,6 [ 1,85; 3]	5,8 [ 2,33; 12,5]	0,96 [ 0,38; 2,26]	1,27 [ 0,93; 1,714]	p=0,0049 Р1–2=1,0 Р1–3=0,04 Р1–4=0,016 Р2–3=0,28 Р2–4=0,26 Р3–4=1,0
Дезоксикортикостерон/прогестерон	1 [ 0,67; 1,43]	3,5625 [ 1,07; 7,5]	0,588 [ 0,19; 1,022]	0,4 [ 0,03; 1] N=21	p=0,12
ДГЭА/17-гидроксипрегненолон	2,38 [ 0,66; 4,36]	1,283 [ 0,367; 2,86]	1,4 [ 0,95; 2,22]	1,315 [ 0,84; 2,47] N=19	p=0,82
17-гидроксипрегненолон/прегненолон	7,64 [ 1,118; 11,575] N=6	1 [ 1; 1,33] N=3	6,63 [ 4,687; 6,93] N=5	2,87 [ 1,26; 4,56] N=10	p=0,12

## ОБСУЖДЕНИЕ

В настоящее время сохраняются трудности в дифференциальной диагностике заболеваний надпочечников в педиатрической практике. В течение последних лет наблюдаются попытки использования мультистероидного профиля для дифференциальной диагностики заболеваний надпочечников.

Одним из самых специфичных показателей для диагностики эндогенного гиперкортицизма по данным исследований мультистероидного профиля у взрослых пациентов оказался непосредственный предшественник кортизола — 11-дезоксикортизол [11–16]. В норме АКТГ стимулирует переход холестерина в прегненолон в митохондриях клеток коры надпочечников, а дальнейший путь, кроме последних ступеней стероидогенеза, происходит в эндоплазматическом ретикулуме [17]. Фермент 11β-гидроксилаза катализирует конверсию 11-дезоксикортизола в кортизол и дезоксикортикостерона в кортикостерон (рис. 1) [18].

В исследование G. Eisenhofer и соавт. были включены 84 пациента с подтвержденным эндогенным гиперкортицизмом (51 пациент с АКТГ-зависимым гиперкортицизмом центрального генеза (кортикотропиномы), 21 пациент с образованиями надпочечников с гиперсекрецией кортизола (кортикостеромы), 12 пациентов с АКТГ-эктопированным синдромом). По результатам исследования мультистероидного профиля уровни предшественников кортизола и альдостерона, 11-дезоксикортизола идезоксикортикостерона, были статистически значимо выше у пациентов с эндогенным гиперкортицизмом любого генеза по сравнению с группой контроля (p<0,0001) [11]. Аналогичные данные были получены в исследовании G. Dalmazi и соавт., вкоторое были включены 302 пациента (98 пациентов с автономной гиперсекрецией кортизола (кортикостерома), 204 пациента с инциденталомой надпочечника), уровни 11-дезоксикортизола и дезоксикортикостерона были статистически значимо вышев кортикостеромах (p=0,023 и 0,047 соответственно) [13]1.

По результатам исследований повышение уровня 11-дезоксикортизола также являлось специфичным для адренокортикального рака (АКР) [14][15]. В исследование D. Taylor и соавт. были включены 48 пациентов (из них 10 пациентов с АКР, 7 — скортикостеромой, с феохромоцитомами/параганглиомами — 15 пациентов, с инциденталомой надпочечника — 16), и уровни 11-дезоксикортизола был статистически значимо выше у пациентов с АКР (p<0,005) [14], а в исследование S. Schweitzer исоавт. были включены 108 пациентов (из них с АКР 42 пациента, с кортикостеромой — 24, с инциденталомой — 42), уровни 11-дезоксикортизола также статистически значимо были выше у пациентов с АКР (p<0,01), как и уровни дезоксикортикостерона (p<0,001) [15].

По результатам исследования анализа мультистероидного профиля у детей, в которое были включены 8 детей (4 пациента с АКР, 4 пациента с доброкачественным образованием надпочечника, у всех пациентов была выявлена гиперандрогения), уровни 11-дезоксикортизола и дезоксикортикостерона были повышены у всех пациентов [16], с послеоперационным восстановлением нормальных значений. По результатам этого исследования авторы статьи делают вывод о возможности использовать уровень 11-дезоксикортизола для диагностики объемных образований надпочечников, но не для дифференциальной диагностики АКР и аденом надпочечников.

В нашей группе пациентов выявлены более высокие уровни 11-дезоксикортизола в группе пациентов с кортикостеромами, но не у пациентов с АКТГ-зависимым гиперкортицизмом. Также выявлено значимое снижение соотношения кортизол/11-дезоксикортизол в группе пациентов с кортикостеромами. Можно предположить, что достоверное повышение уровня 11-дезоксикортизола при АКТГ-независимом гиперкортицизме по сравнению с АКТГ-зависимым вариантом, при сопоставимости уровня кортизола в этих группах, свидетельствует о возможном механизме субстратзависимого подавления активности фермента 11β-гидроксилазы в отсутствие стимуляции АКТГ.

По результатам нашего исследования у пациентов с кортикостеромами также отмечались статистически значимо сниженные уровни 17-гидроксипрегненолона и ДГЭА по сравнению с группой пациентов с инциденталомами надпочечников (р=0,0026и р=0,0047 соответственно), и аналогичная тенденция по сравнению с группами пациентов с преждевременным адренархе (р=0,057 и р=0,052 соответственно), которая, однако, не достигала статистической достоверности, но не с группой пациентовс АКТГ-зависимым гиперкортицизмом (р=0,21 и р=0,14 соответственно). По данным литературы, у взрослых пациентов с гиперкортицизмом надпочечникового генеза выявлялись статистически значимо более низкие уровни андрогенов (андростендиона, ДГЭА и ДГЭА-С) по сравнению как с другими вариантами гиперкортицизма, так и с инциденталомами [11][13]. Снижение уровня андрогенов при гиперкортицизме надпочечникового генеза связывают с низким уровнем АКТГ при данной патологии, который является главным стимулирующим фактором для всех ферментов стероидогенеза. Можно предполагать, что отсутствие стимуляции сетчатой зоны коры надпочечников в условиях подавленного АКТГ приводит в первую очередь к снижению активности 17α-гидроксилазы. Действительно в нашем исследовании соотношение 17-гидроксипрегненолон/прегненолон оказалось статистически значимо ниже в группе пациентов с кортикостеромами по сравнению с другими группами, что может свидетельствовать о низкой 17α-гидроксилазной активности. При этом статистически значимых различий соотношения ДГЭА/17-гидроксипрегненолон между группами не выявлено, что может свидетельствовать о сохранной 17,20-лиазной активности фермента CYP17.

Еще одной особенностью, характеризующей активность ферментов стероидогенеза у пациентов с АКТГ-независимым гиперкортицизмом, является тенденция к повышению активности 21-гидроксилазы.

Таким образом, по результатам исследования мультистероидного профиля у детей с разными заболеваниями надпочечников в нашем исследовании выявлены особенности, характерные для кортикостером, в отличие как от АКТГ-зависимого гиперкортицизма, так и от инциденталом надпочечника. Эти особенности заключаются в повышении уровня 11-дезоксикортизола и снижении уровня 17-гидроксипрегненолона и ДГЭА. Эти особенности стероидного профиля кортикостером могут служить дополнительным дифференциально-диагностическим критерием.

## ОГРАНИЧЕНИЯ ИССЛЕДОВАНИЯ

Исследование является ретроспективным, поэтому нельзя исключить историческое смещение в оценке лабораторных показателей. С ретроспективным дизайном связано и значительное количество пропусков в данных. Статистически значимые различия в некоторых группах не вычислены в связи с малым количеством пациентов в группах. Пациенты с АКР не были включены в наше исследование в связи с маленьким количеством данных.

## ЗАКЛЮЧЕНИЕ

В связи с редкой встречаемостью объемных образований надпочечников и эндогенного гиперкортицизма в детской популяции сохраняются трудности в диагностике и дифференциальной диагностике данных патологий. Для постановки диагноза сохраняется потребность в проведении большого количества лабораторных методов исследований с сохранением высокой частоты как ложноположительных, так и ложноотрицательных результатов. По результатам нашей работы исследование мультистероидного профиля может использоваться в качестве дополнительного метода дифференциальной диагностики пациентов с образованиями надпочечников с и без гормональной гиперпродукции (кортикостеромы и инциденталомы надпочечников). Необходим дальнейший поиск стероидных маркеров для дифференциальной диагностики различных заболеваний надпочечников у детей.

## ДОПОЛНИТЕЛЬНАЯ ИНФОРМАЦИЯ

Источник финансирования. Исследование выполнено в рамках государственного задания «Молекулярно-генетические, масс-спектрометрические и иммуногистохимические маркеры в персонализации диагностики и лечении гиперкортицизма у детей», регистрационный номер АААА-А20-120011790183-5.

Конфликт интересов. Все авторы декларируют отсутствие явных и потенциальных конфликтов интересов, связанных с публикацией настоящей статьи.

Участие авторов. Все авторы одобрили финальную версию статьи перед публикацией, выразили согласие нести ответственность за все аспекты работы, подразумевающую надлежащее изучение и решение вопросов, связанных с точностью или добросовестностью любой части работы.

1. В данном исследовании отсутствуют данные о применении поправки на множественные сравнения.
